# An insight into tetracycline photocatalytic degradation by MOFs using the artificial intelligence technique

**DOI:** 10.1038/s41598-022-10563-8

**Published:** 2022-04-22

**Authors:** Majedeh Gheytanzadeh, Alireza Baghban, Sajjad Habibzadeh, Karam Jabbour, Amin Esmaeili, Ahmad Mohaddespour, Otman Abida

**Affiliations:** 1 Surface Reaction and Advanced Energy Materials Laboratory, Chemical Engineering Department, Amirkabir University of Technology (Tehran Polytechnic), Tehran, Iran; 2 Chemical Engineering Department, Amirkabir University of Technology (Tehran Polytechnic), Mahshahr Campus, Mahshahr, Iran; 3 College of Engineering and Technology, American University of the Middle East, Kuwait City, Kuwait; 4 Department of Chemical Engineering, School of Engineering Technology and Industrial Trades, College of the North Atlantic-Qatar, Doha, Qatar

**Keywords:** Environmental sciences, Materials science, Nanoscience and technology

## Abstract

Tetracyclines (TCs) have been extensively used for humans and animal diseases treatment and livestock growth promotion. The consumption of such antibiotics has been ever-growing nowadays due to various bacterial infections and other pathologic conditions, resulting in more discharge into the aquatic environments. This brings threats to ecosystems and human bodies. Up to now, several attempts have been made to reduce TC amounts in the wastewater, among which photocatalysis, an advanced oxidation process, is known as an eco-friendly and efficient technology. In this regard, metal organic frameworks (MOFs) have been known as the promising materials as photocatalysts. Thus, studying TC photocatalytic degradation by MOFs would help scientists and engineers optimize the process in terms of effective parameters. Nevertheless, the costly and time-consuming experimental methods, having instrumental errors, encouraged the authors to use the computational method for a more comprehensive assessment. In doing so, a wide-ranging databank including 374 experimental data points was gathered from the literature. A powerful machine learning method of Gaussian process regression (GPR) model with four kernel functions was proposed to estimate the TC degradation in terms of MOFs features (surface area and pore volume) and operational parameters (illumination time, catalyst dosage, TC concentration, pH). The GPR models performed quite well, among which GPR-Matern model shows the most accurate performance with R^2^, MRE, MSE, RMSE, and STD of 0.981, 12.29, 18.03, 4.25, and 3.33, respectively. In addition, an analysis of sensitivity was carried out to assess the effect of the inputs on the TC photodegradation by MOFs. It revealed that the illumination time and the surface area play a significant role in the decomposition activity.

## Introduction

In recent decades, water pollution and wastewater treatment have become a global concern^1,2^. Notably, the release of wastewater having antibiotics can lead to severe environmental and human health issues^3^. The discharged antibiotics in water may contribute to the food chain and enter the living things in time. Therefore, they are known as a category of emerging organic pollutants^4^. Among the antibiotics, tetracycline (TC) can be found in different water environments, from underground waters to surface waters. It is raised from its rampant overuse, non-biodegradability, and easy dissolution in water^5,6^. Indeed, it is challenging to degrade TC in natural conditions due to its aromatic rings with stable molecular structure^7^. The TC residual can bring antibiotic-resistant bacteria and genes into the ecological system^8^. Therefore, it is vital and urgent to control the TC concentration level in the environment.

For this purpose, many effective methods have been examined to eliminate the TCs pollution, such as adsorption^9,10^, ozonation^11^, ion-exchange^12^, electrocoagulation^13^, and biological methods^14^. Nevertheless, there are undeniable shortcomings with these approaches. For example, through the physical separation methods without mineralization, the TCs need post-treatments to be degraded after separation^15^. The biological degradation procedures show low removal efficiency due to their antibacterial nature^16^. Moreover, the high treatment cost or generating of carcinogenic chlorinated intermediates limit the use of traditional chemical oxidation routes such as ozonation or chlorine oxidation^17^. Lately, semiconductor photocatalysis, an advanced oxidation process (AOP), has attracted massive attention due to its low cost and significant efficiency^18–20^. It is based on the producing hydroxyl radical (^•^OH), a highly active agent that degrades TC to a more minor or inorganic substance^18^. Selecting the appropriate photocatalyst is crucial to achieving excellent efficiency in photocatalysis.

Metal–organic frameworks (MOFs), a new class of porous crystalline substances, have received significant interest as a porous material in different processes^21–25^. The MOFs are one-, two-, or three-dimensional network structures constructed by metal ions/-clusters coordinated to organic ligands (linkers). The most exciting property of MOFs is their adaptable framework which can be modified by changing the linkers. Thus, a significantly porous material with excellent surface area could be provided, which benefits surface phenomena^26^. Furthermore, some MOFs with semiconductor properties have been utilized as photocatalysts^27,28^. Several studies have reported the effective performance of MOFs and their derivatives in the photodegradation of TCs^27,29,30^.

Several factors influence photocatalysis efficiency, such as the pollutant concentration, the photocatalyst dosage, and the solution pH^31–33^. Extensive research in the photocatalysis area has been conducted to find the optimum parameters, making it possible to gather tremendous data sets^34–36^. However, a complicated nonlinear relationship exists between the effective parameters and the degradation efficiency (the output). On the other hand, conducting extra different experiments to figure out the relationship is very time-consuming and costly, and also, it is not possible to consider all the influential experimental variables. Therefore, to save cost and experimental time, computational modeling is utilized as an alternative to assess the photocatalytic degradation of organic pollutants. On the other hand, as the complexity of the case study increases, the CPU-consuming and calculation time of the simulation increase significantly. This matter inspired the authors to use alternative methods such as machine learning (ML) techniques.

The robust ML methods can recognize the relationship between the different parameters of the process and their corresponding outcomes through passing the requirement to solve theoretical equations^37,38^. The prognostication of nonlinear and complicated systems performance, high reliability, recognizing each influencing parameter's effect, and finding their optimal are some of the ML approaches advantages^39–49^. Several researchers have applied different ML methods to anticipate the contaminant photocatalytic removal based on different operational variables as inputs^37–39,50–53^.

In this work, for the first time, an effort is performed to use the gaussian process regression (GPR) model with four kernel functions to estimate the TC degradation by MOFs photocatalyst in the photocatalytic wastewater treatment process. A large experimental data bank of TC photodegradation by different MOFs is gathered for this aim. The operational parameters of TC concentration, photocatalyst amount, solution pH, irradiation time, and MOFs structural parameters of specific surface area and pore volume are chosen as input variables. Various statistical analyses were performed to study the developed GPR models. Also, sensitivity analysis was applied to show the most influential parameter(s) in the TC removal by MOFs.

## Methodology

### Data collection

An experimental data set with the total number of 374 of TC photocatalytic degradation using MOF as the photocatalyst, were collected from the reported studies^54–63^ and presented in the supplementary information. The assessed MOFs are MIL-88A, MIL-101(Fe), MIL-125(Ti), Cu-TCPP, PI/UiO-66-NH_2_, AgI/UiO-66-NH_2_, PANI/MIL-100(Fe), In_2_S_3_/MIL-125(Ti), Ag_2_S/MIL-53(Fe), and WO_3_/GO/UiO-66. Also, the catalyst dosage (g/L), the initial concentration of TC (mg/L), the solution pH, and the irradiation time (min), along with MOFs’ structural features of pore volume (cm^3^/g) and specific surface area (m^2^/g), are the input parameters. To establish the most accurate model, 80% of the data set was randomly separated as a training set while the rest (20%) was considered testing data set to evaluate the preciseness of the developed model. Model accuracy examination was quantified through calculation of statistical parameters such as R^2^, mean relative error (MRE), root-mean-square error (RMSE), mean-square error (MSE), and the standard deviation (STD), which are given as follows:

1 $${R}^{2}=1-\frac{{\sum }_{i=1}^{n}{\left[{x}_{i}^{predicted}-{x}_{i}^{experimental}\right]}^{2}}{{\sum }_{i=1}^{n}{\left[{x}_{i}^{predicted}-{x}_{m}\right]}^{2}}$$

2 $$STD=\sqrt{\sum_{i=1}^{n}\frac{{({x}_{i}^{predicted}-{x}_{m})}^{2}}{n}} $$

3 $$MSE=\frac{1}{n}\sum_{i=1}^{n}{\left({x}_{i}^{predicted}-{x}_{i}^{experimental}\right)}^{2} $$

4 $$RMSE=\sqrt{\frac{\sum_{i=1}^{n}{\left({x}_{i}^{predicted}-{x}_{i}^{experimental}\right)}^{2}}{n}}$$

5 $$MRE=\frac{1}{n}\sum_{i=1}^{n}\frac{\left|{x}_{i}^{predicted}-{x}_{i}^{experimental}\right|}{{x}_{i}^{experimental}} $$

### Gaussian process regression (GPR)

GPR model is an effective nonparametric and probabilistic supervised ML method capable of modeling nonlinear complicated issues^64^. It utilizes the Gaussian process to perform regression. One of the main attractive features of this approach is its flexible algorithm in the description of the uncertainty^65^.

In general, for GPR modeling: suppose $T={\left\{{x}_{T.i}.{y}_{T.i}\right\}}_{i=1}^{n}$ and $L={\left\{{x}_{L.i}.{y}_{L.i}\right\}}_{i=1}^{n}$ are randomly chosen test and training data sets, respectively, where x and y are the input and the outcome variables. The GPR modeling starts from the following equation:

6 $${y}_{L.i}=f\left({x}_{L.i}\right)+{\varepsilon }_{L.i} i=1.2.3.... .n$$

7 $$\varepsilon \sim N(0. {\sigma }_{noise}^{2}{I}_{n})$$

where *x*_*L*_ and *y*_*L*_ indicate the independent variables and the outcomes of the training data points, respectively. Also, $\varepsilon$ is the observation noise, σ^2^_noise_ represents the variance of the noise, and *I*_*n*_ is the unit array. Similarly, we can write for test data set:

8 $${y}_{T.i}=f\left({x}_{T.i}\right)+{\varepsilon }_{T.i} i=1.2.3.... .n$$

The symbols have the same definition as mentioned so far but for the test data set. Therefore, the Gaussian noise model connects every calculated *y* to the considered *f(x)* function. According to the GPR model, *f(x)* is a random function which can be determined by its corresponding mean *m(x)* and covariance *k(x, x′)* (also called kernel) functions.

9 $$f\left({x}_{L.i}\right) \sim GP(m\left(x\right). k(x.{x}^{{\prime}})) $$

The *m(x)* can be determined through utilizing the explicit basis functions, but usually, it is considered as zero in the simplified calculations^66,67^:

10 $$f\left({x}_{L.i}\right) \sim GP(0. k(x.{x}^{{\prime}}))$$

Combination of Eqs. (6) and (10) give the distribution of *y*:

11 $$y \sim N(0. k\left(x.{x}^{{\prime}}\right)+{\sigma }_{noise}^{2}{I}_{n}) $$

According to the mentioned parameters and definitions, we have:

12 $$\left[\begin{array}{c}\underset{{f}_{L}}{\to }\\ \underset{{f}_{T}}{\to }\end{array}\right] \sim N\left(0. \left[ \begin{array}{cc}k\left({x}_{L}.{x}_{L}\right)& k\left({x}_{L}.{x}_{T}\right)\\ k\left({x}_{T}.{x}_{L}\right)& k\left({x}_{T}.{x}_{T}\right)\end{array}\right]\right)$$

13 $$\left[\begin{array}{c}\underset{{\varepsilon }_{L}}{\to }\\ \underset{{\varepsilon }_{T}}{\to }\end{array}\right] \sim N\left(0. \left[\begin{array}{cc}{\sigma }_{noise}^{2}{I}_{n}& 0\\ 0& {\sigma }_{noise}^{2}{I}_{n}\end{array}\right]\right) $$

The Gaussian expression is obtained from the summation of the recent two equations:

14 $$\left[\begin{array}{c}\underset{{y}_{L}}{\to }\\ \underset{{y}_{T}}{\to }\end{array}\right] \sim N\left(0. \left[\begin{array}{cc}k\left({x}_{L}.{x}_{L}\right)+{\sigma }_{noise}^{2}{I}_{n}& k\left({x}_{L}.{x}_{T}\right)\\ k\left({x}_{T}.{x}_{L}\right)& k\left({x}_{T}.{x}_{T}\right)+{\sigma }_{noise}^{2}{I}_{n}\end{array}\right]\right)$$

The conditioning rule of Gaussians can be used to achieve the distribution of the *y*_*T*_ :

15 $$\left({y}_{T}|{y}_{L}\right)\sim N\left({\mu }_{T}.{\Sigma }_{T}\right) $$

16 $${\Sigma }_{T}=k\left({x}_{T}.{x}_{T}\right)=k\left({x}_{T}.{x}_{T}\right)+{\sigma }_{noise}^{2}{I}_{n}-{k\left({x}_{T}.{x}_{L}\right)\left(k\left({x}_{L}.{x}_{L}\right)+{\sigma }_{noise}^{2}{I}_{n}\right)}^{-1}k({x}_{L}.{x}_{T})$$

17 $${\mu }_{T}=m\left(\underset{{y}_{T}}{\to }\right)=k\left({x}_{T}.{x}_{L}\right){\left(k\left({x}_{L}.{x}_{L}\right)+{\sigma }_{noise}^{2}{I}_{n}\right)}^{-1} \underset{{y}_{T}}{\to }$$

where the *Σ*_*T*_ and *μ*_*T*_ are the covariance and the mean value, respectively. Selecting a kernel function, including a symmetric invertible matrix, can influence the strength and the robustness of the prediction ability of the final GPR model. Accordingly, four different kernel functions of Exponential, Squared exponential, Matern, and Rational quadratic are selected to find the best one. The selected kernel functions are presented as follow:

- Exponential kernel function:
  18 $${k}_{E}\left(x.{x}^{{\prime}}\right)={\sigma }^{2}exp\left(-\frac{x-{x}^{{\prime}}}{\mathcal{l}}\right)$$
- Squared Exponential kernel function:
  19 $${k}_{SE}\left(x.{x}^{{\prime}}\right)={\sigma }^{2}exp\left(-\frac{x-{x}{{^{\prime}} 2}}{{\mathcal{l}}^{2}}\right) $$
- Matern kernel function:
  20 $${k}_{M}\left(x.{x}^{{\prime}}\right)={\sigma }^{2}\frac{{2}^{1-v}}{\Gamma \left(v\right)}{\left(\sqrt{2v}\frac{x-{x}^{{\prime}}}{\mathcal{l}}\right)}^{v}{K}_{v}\left(\sqrt{2v}\frac{x-{x}^{{\prime}}}{\mathcal{l}}\right)$$
- Rational quadratic kernel function:
  21 $${k}_{RQ}\left(x.{x}^{{\prime}}\right)={\sigma }^{2}{\left(1+\frac{x-{x}{{^{\prime}} 2}}{2a\mathcal{l}}\right)}^{-a}$$

Where α > 0, σ, σ^2^, and ℓ represent scale-mixture, the amplitude, the variance, and the length scale, respectively. Additionally, the K_v_, Γ, and *v* indicate the modified Bessel function, the gamma function, and a positive parameter, respectively.

### Data set outlier detection

Outlier or suspected data has different behavior compared to the rest of the data points. These data often arise from the experiment or instrumental errors. The detection of suspected data in the data set is necessary to avoid the wrong examination of the established model and make it more efficient. To do so, Leverage method was used in which the Hat matrix is defined as follows:

22 $$H=U{({U}^{T}U)}^{-1}{U}^{T} $$

U is an i*j dimensional matrix, and i and j represent the parameters number and the training data number, respectively. The preciseness of the data set is assessed by plotting the standardized residuals versus Hat values, called William’s plot. In this diagram, a reliable region is defined which the data out of it are suspected data. The limited area between standardized residuals of −3 to 3 and Hat values of 0 to critical leverage limit, is considered as the reliable zone. The critical leverage limit is calculated as follows^68,69^:

23 $${H}^{*}=\frac{3(j+1)}{i}$$

**Figure 1:**
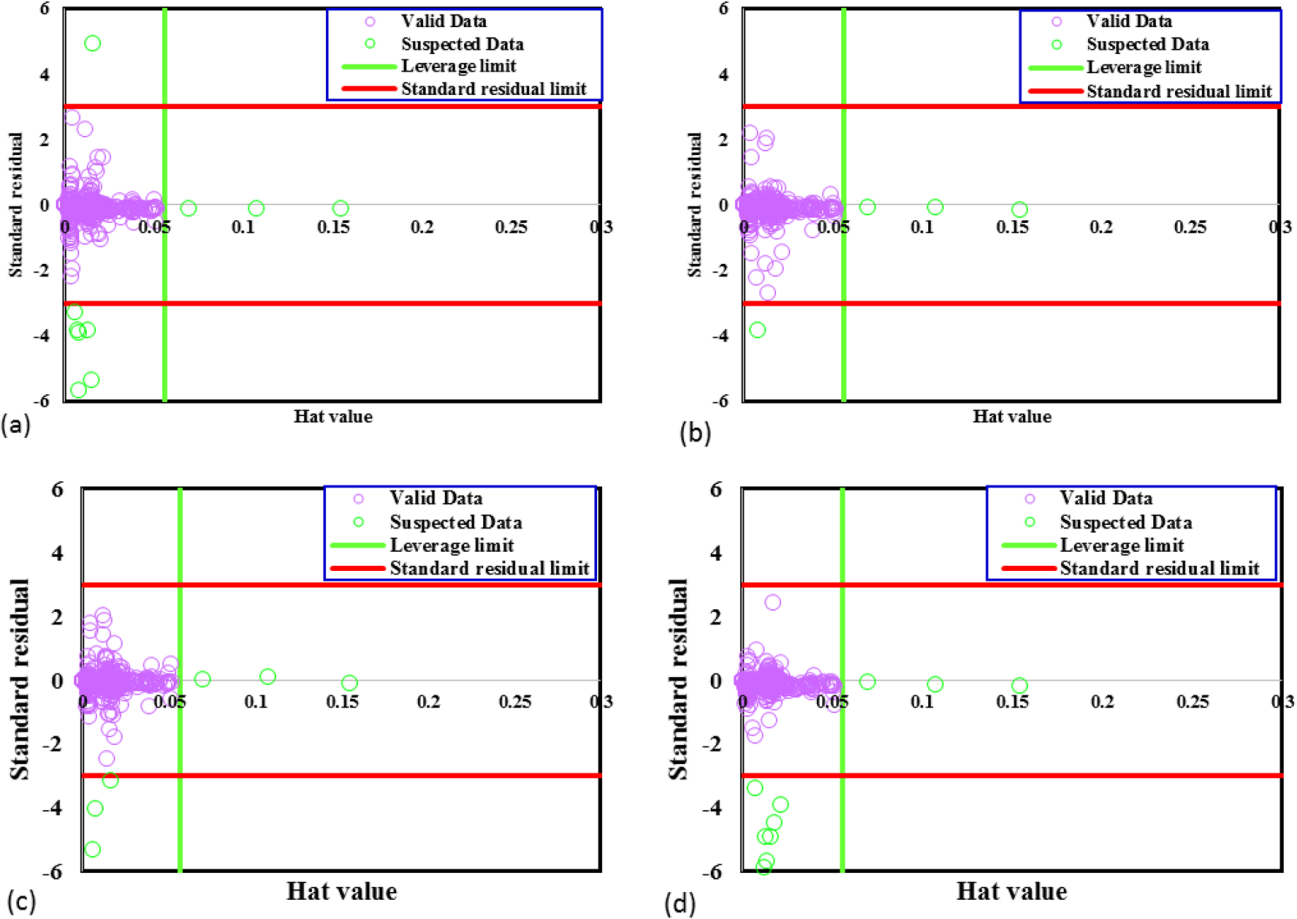
Detection of outliers for GPR model containing kernel function of **(a)** matern, **(b)** exponential, **(c)** squared exponential, **(d)** rational quadratic.

According to William’s plot of the TC removal data bank (Fig. 1), most of the used data are reliable. In detail, from 374 data points, the number of outliers are only 10, 4, 6, and 10 for GPR-Matern, GPR-Exponential, GPR-Squared Exponential, and GPR-Rational quadratic models, respectively.

## Results and discussion

### Sensitivity analysis

Determining the effect of various operational variables on the outcome is very important for researchers and engineers to suggest an accurate model. To attain this aim, an analysis of sensitivity has been performed through which the relevancy factor of each input variable was calculated as follows^40^:

24 $$r=\frac{{\sum }_{i=1}^{n}\left({X}_{k.i}-{\overline{X} }_{k}\right)\left({Y}_{i}-\overline{Y }\right)}{\sqrt{{\sum }_{i=1}^{n}{\left({X}_{k.i}-{\overline{X} }_{k}\right)}^{2}{\sum }_{i=1}^{n}{\left({Y}_{i}-\overline{Y }\right)}^{2}}}$$

where ${X}_{k.i}$ and ${Y}_{i}$ stand the ‘k’ th input and ‘i’ th output while ${\overline{X} }_{k}$ and $\overline{Y }$ indicate the average values of input and outputs, respectively.

**Figure 2:**
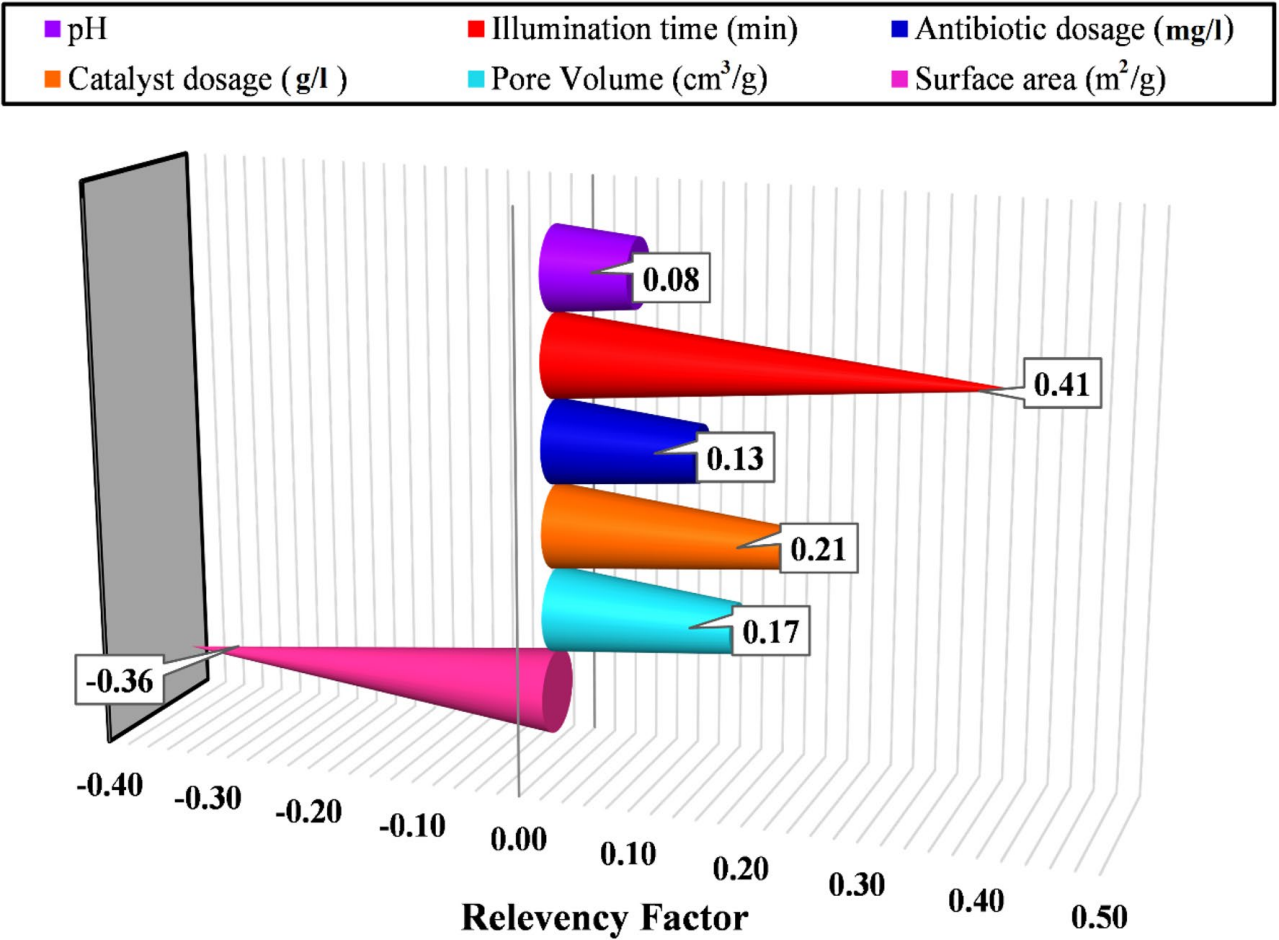
Sensitivity analysis of the input operational variables for TC photodegradation by various MOFs.

Each input with a more considerable r value has a greater effect on the output quantity. The negative values denote that the corresponding parameter negatively affects the model outcome and vice versa. As depicted in Fig. 2, the illumination time is the most influential parameter in the TC degradation on various MOFs. As the time of the process increase, the degradation improves. Indeed, time is the only parameter which alters along with the degradation percentage simultaneously during the experiment.

In the case of solution pH, it affects the overall charge of the catalyst and pollutant, and consequently, the adsorption of pollutants on the surface and degradation could be influenced. Thus, it is not necessarily correct that the pollutant degrades faster at higher pH because several MOFs with various structures have different responses to pH^51^. For instance, Zhang et al.^57^ reported that the pH  3 is the best value to degrade TC on MIL-88A, while Pan et al.^61^ claimed that the TC was degraded over AgI/UiO-66-NH_2_ more rapidly in pH  4.3.

The surface area is the second most influential parameter with a negative effect. In this case, we cannot interpret directly without considering the following factors. First, the researchers often used MOFs composed with another compound, fabricating composites to enhance the photodegradation efficiency. In some cases, it declines the surface area and no/small change in pore sizes. In other words, the MOF pores got blocked by introducing the second photocatalysts. For example, the 53% TC degradation by UiO-66 with 856.61 m^2^/g increased to 86% after composite with WO_3_/graphene oxide while the surface area decreased to 379.51 m^2^/g ^60^. Secondly, about 60% of the data points (218 out of 374 data points) have a lower surface area than the mean value of the data bank. It indicates that most data points are obtained from MOFs with low surface area. Indeed, lower data points with high surface area and excellent degradation contribute to the sensitivity analysis of the surface area with the negative sign. Besides, significant changes in MOFs surface area (from 15.95 to 1548.3 m^2^/g) result in the severe effect of this parameter on the outcome.

Furthermore, input parameters such as catalyst dosage and antibiotic concentration have critical value to influence positively in the photocatalytic experiments. As the catalyst dosage increases, a large number of charge carriers are generated, increasing the degradation efficiency. It is until a certain amount because high amount of catalyst avoids the light from reaching the inner environment of the solution. The extra amount of TC leads to a competition between the TC and reaction-intermediates molecules for being absorbed on the catalyst surface. Thus, the two last matters cause a decrease in the TC degradation efficiency^38^. Considering the above discussion, the results of sensitivity analysis must interpret with enough knowledge of the under-study phenomenon. Here, some parameters of chemical features of the MOFs and the contaminant or photocatalytic properties such as the recombination rate of the photogenerated charge carriers and catalyst light absorbance were not considered while could be determining factors. Therefore, as Ayodele et al.^53^ reported, the importance of the parameters varies based on the photocatalytic process conditions.

### Modeling results and validation

In order to examine the anticipation ability of the suggested GPR models for TC photodegradation by MOFs, several assessments have been carried out and presented. The validation assessments are provided in two main approaches: statistical matching factors and graphical comparison plots. The statistical parameters indicate how much the actual and predicted TC removal values match. These parameters are listed in Table 1 for the training, testing, and total data sets. For training data, the R^2^ values of 0.980, 0.979, 0.973, and 0.967 are achieved for the developed GPR models with kernel functions of Matern, Exponential, Squared Exponential, and Rational Quadratic, respectively. Also, their corresponding error values of MRE, MSE, RMSE, and STD are low, confirming that they have trained the data with adequate accuracy. In addition to the training data, the capability of the established GPR models in anticipation of the new (unseen) TC photodegradation data points is vital. Thus, the models have also been evaluated for the testing data set. As presented in Table 1, the GPR model with Matern kernel function shows the most accurate performance for the photocatalytic degradation of TC by MOFs (R^2^, MRE, MSE, RMSE, and STD are 0.981, 12.29, 18.03, 4.25, and 3.33, respectively). Note that the GPR-Exponential model also has acceptable results close to the GPR-Matern model.

**Table 1:** The statistical parameters of suggested GPR models.

Model	Dataset	R^2^	MRE (%)	MSE	RMSE	STD
GPR matern	Train	0.980	12.20	16.13	4.02	3.21
	Test	0.981	12.29	18.03	4.25	3.33
	Total	0.980	12.22	16.60	4.25	3.24
GPR Exp	Train	0.979	15.54	17.09	4.13	3.15
	Test	0.977	11.81	21.55	4.64	3.34
	Total	0.979	14.61	18.20	4.64	3.20
GPR SExp	Train	0.973	14.56	22.47	4.74	3.52
	Test	0.970	19.92	28.40	5.33	3.92
	Total	0.972	15.89	23.95	5.33	3.63
GPR rational quadratic	Train	0.967	19.58	27.20	5.22	3.86
	Test	0.969	28.27	28.93	5.38	4.03
	Total	0.967	21.74	27.63	5.38	3.90

**Figure 3:**
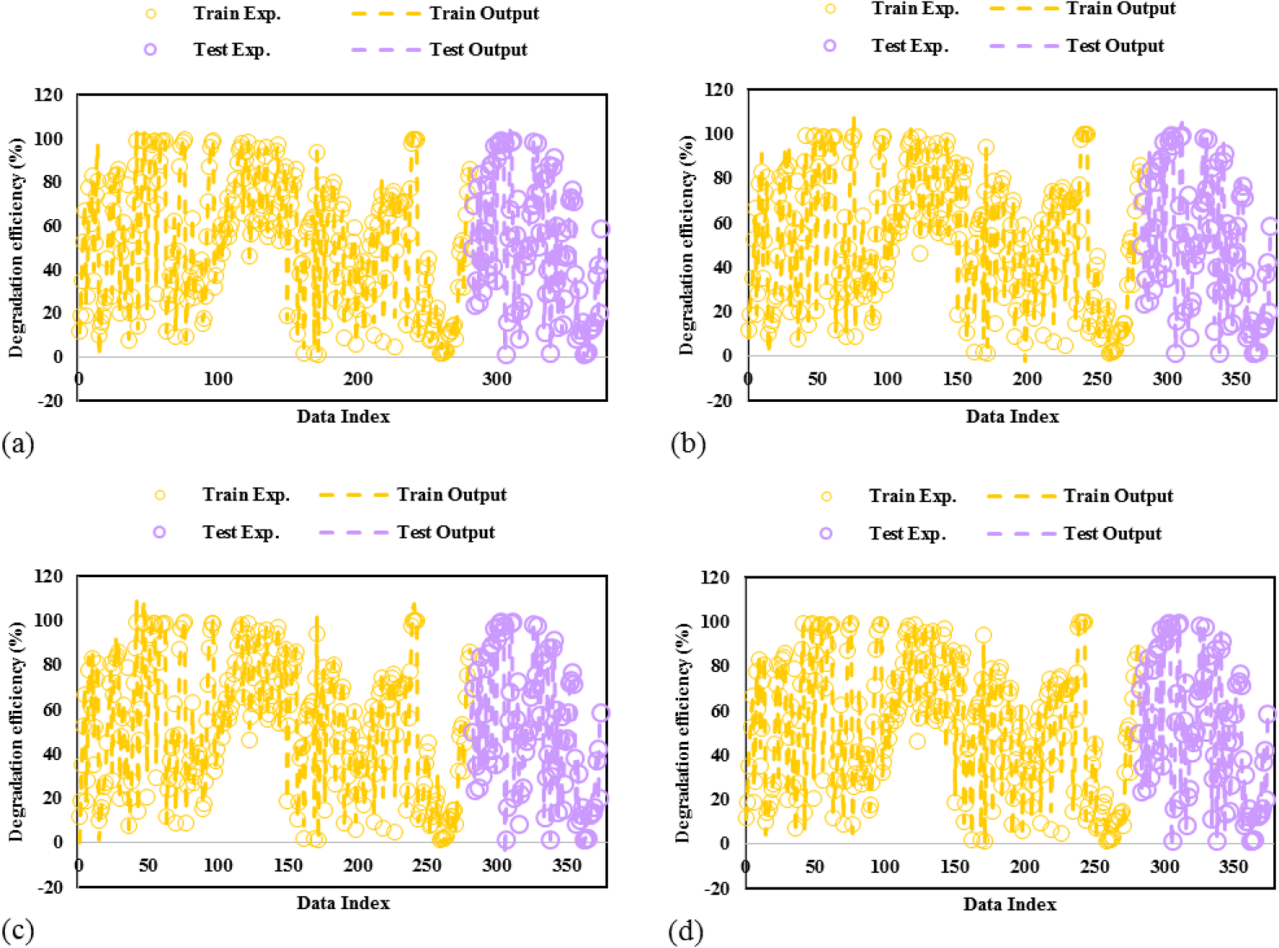
Comparison of actual and predicted values for GPR model with kernel function of **(a)** matern, **(b)** exponential, **(c)** squared exponential, **(d)** rational quadratic.

The experimental and predicted values of TCs photodegradation are simultaneously depicted in Fig. 3. As shown, the predicted amounts of TC photocatalytic degradation by MOFs have excellent agreements with the actual amounts. Indeed, all the proposed models' results could follow the experimental data points in training and testing data sets. Thus, the developed GPR models present reliable anticipation performance for the TC-MOF photodegradation system.

**Figure 4:**
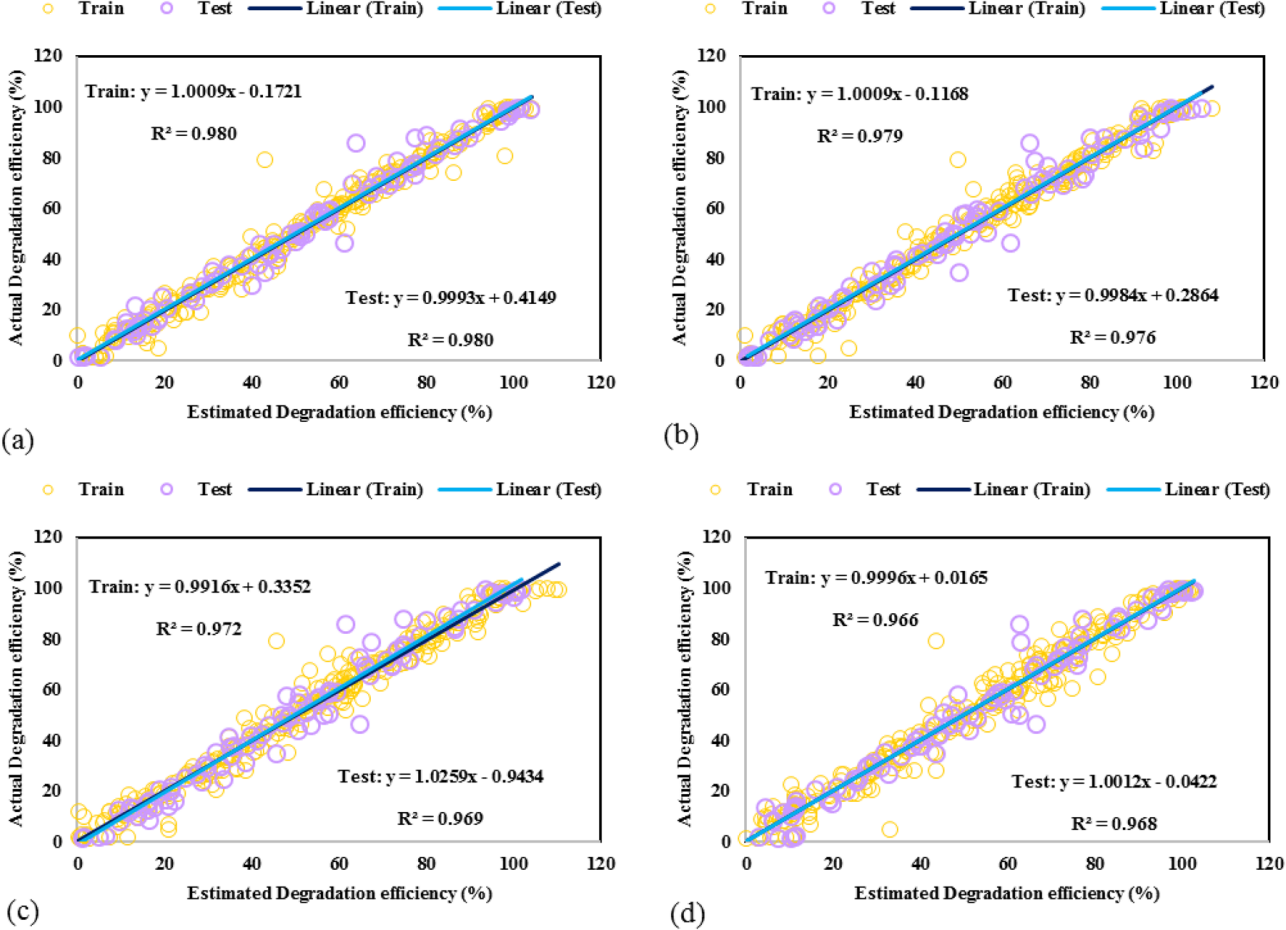
Cross plots for GPR model containing kernel function of **(a)** exponential, **(b)** matern, **(c)** squared exponential, **(d)** rational quadratic.

In addition, the actual TC photodegradation efficiencies versus the GPR models' estimated ones are plotted in Fig. 4, namely, cross plot. In this plot, the bisector line in the first quarter is the prediction accuracy criterion. The closer data points to the bisector line, the more accuracy the suggested models obtain. As shown, for all the GPR models, particularly the GPR-Matern, the predicted data placed on the actual data where the fitting line of both data sets have near unit R^2^ values (R^2^ = 0.98 in the GPR-Matern model). Therefore, it is confirmed that the GPR models can predict the experimental data set in this study.

**Figure 5:**
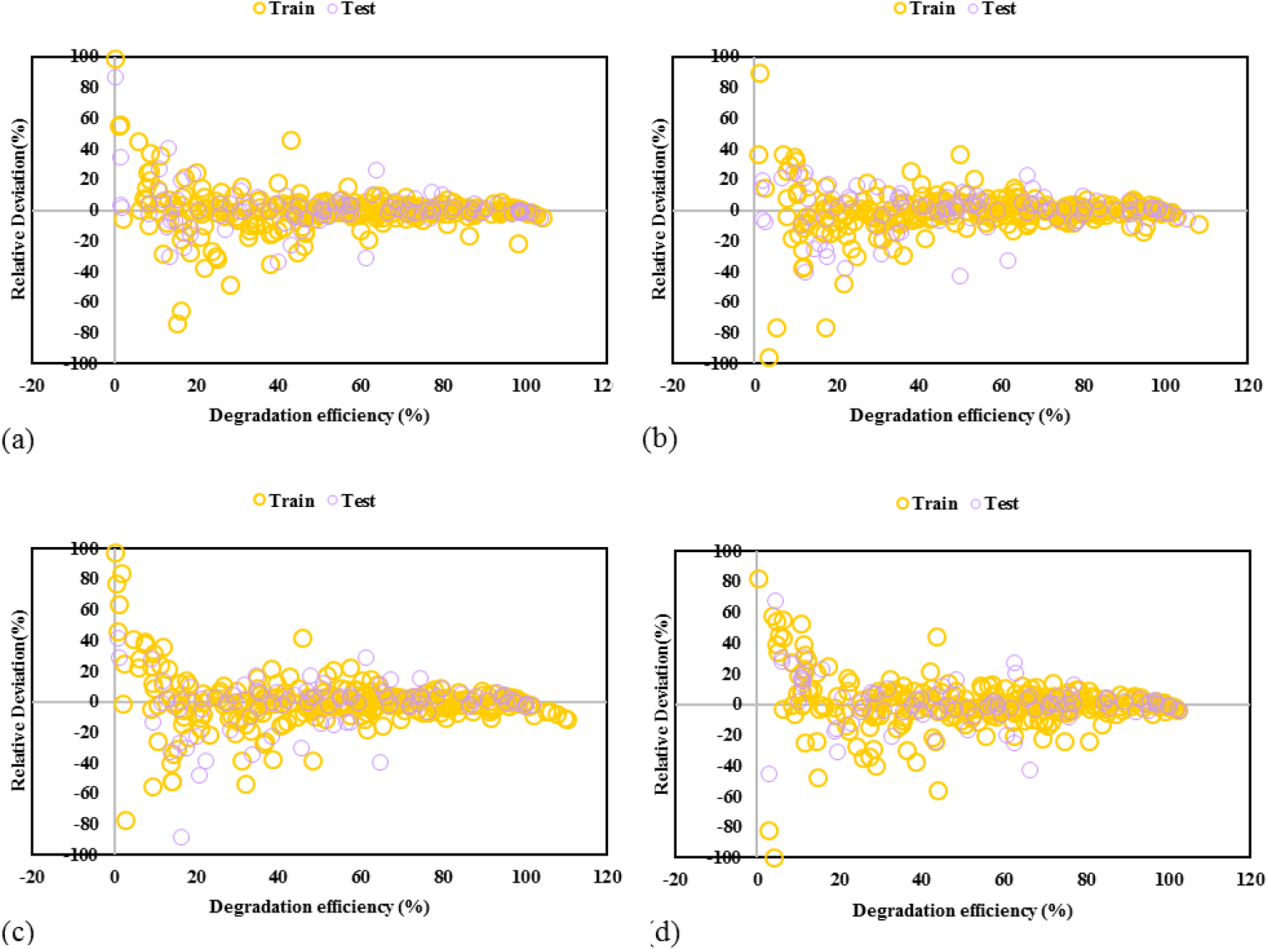
Comparison of actual and model predicted values for GPR model containing kernel function of **(a)** matern, **(b)** exponential, **(c)** squared exponential, **(d)** rational quadratic.

In the next step of models’ results validation, the percentage of relative deviation between actual and model estimated TC removal is calculated and depicted in Fig. 5. For all the GPR models, the absolute relative deviation pints are mostly lower than 30%, while for GPR with Matern kernel function, they are lower than 20%. Also, GPR-Matern gives the minimum mean relative error of 12.22% compared to GPR- Exponential (14.61%), GPR-Square exponential (15.89%), and GPR-Rational quadratic (21.74%).

## Conclusion

In this study, the machine learning method of Gaussian process regression (GPR) model with four kernel functions of Exponential, Squared exponential, Matern, and Rational quadratic were investigated to anticipate the TC photodegradation from wastewater by MOFs. The MOFs features (surface area and pore volume) and operational parameters (illumination time, catalyst dosage, TC concentration, pH) were chosen as the input parameters. The established models prognosticated the experimental TC degradation very well. The GPR-Matern model shows the best performance with R^2^, MRE, MSE, RMSE, and STD of 0.981, 12.29, 18.03, 4.25, and 3.33, respectively. The analysis of sensitivity specifies that the illumination time is the most influential parameter in TC photodegradation by MOFs. The MOFs surface area was found as the second determining input in the TC-MOFs photocatalytic systems. The presented discussions in the current study could make it a supportive report for the engineers and researchers dealing with TCs contaminant elimination or control technologies.

## Supplementary Information

Supplementary Information.

## Data Availability

All data generated or analysed during this study are included in this published article [and its supplementary information files].
